# Assessment of quality and relevance of curricula development in health training institutions: a case study of Kenya

**DOI:** 10.1186/s12960-015-0048-9

**Published:** 2015-08-13

**Authors:** Hazel M Mumbo, Joyce W Kinaro

**Affiliations:** IntraHealth International Inc. FUNZOKenya Project, Nairobi, Kenya

**Keywords:** Kenya, Health workforce capacity, Training institutions, Bottlenecks, Curricula review

## Abstract

The World Health Organization lists Kenya among African countries experiencing health workforce crisis catalysed through immigration, underproduction, inconsistent quality of production and unequal distribution. Strengthening health training institutions to increase production of high-quality health workers is acknowledged as a measure to mitigate the crisis.

IntraHealth International’s USAID-funded FUNZOKenya Project (2012–2017) undertook an assessment to identify the bottlenecks to increasing the number and quality of pre-service graduates in Kenya. The assessment, a cross-sectional descriptive study, collected data through structured respondent interviews among faculty, students in health training institutions, key informants and desk review. The assessment purposively selected 14 institutions from 18 institutions identified for initial collaboration with the project towards strengthening health workforce training. The statistical package for social sciences (SPSS) application helped analyse quantitative data and quotes used to illustrate perceptions on the quality of curricula.

The findings revealed major gaps in quality and adequacy of curricula in the training institutions. A national standard framework to guide curricula review process is lacking. Further, curricula did not adequately prepare students for clinical placement, as most failed to directly respond to national health needs. The study recommended reviews of curricula to ensure their responsiveness to emerging issues in the health sector, the formation of curriculum committees to review curricula, development of official curricula review standards and an integrated mechanism to disseminate policies and guidelines.

**Electronic supplementary material:**

The online version of this article (doi:10.1186/s12960-015-0048-9) contains supplementary material, which is available to authorized users.

## Background

Strengthening of health training institutions to increase the quality and quantity of health workers produced is critical to mitigating global human resources for health crisis and ensuring access to universal quality health coverage. In sub-Saharan Africa, the health workforce crisis is fueled by mass poverty, uneven economic growth and political instability [1]. According to the World Health Organization (WHO) [2], health professional schools as the main channel through which health workers enter the labour market are a key element in addressing the health workforce crisis. However, many health professional institutions are underfunded and operate inefficiently, consequently contributing to the challenge of de-escalation of qualified health workforce production.

According to the Institute of Medicine (US) Committee on Conflict of Interest in Medical Research, Education, and Practice [3], “if the learning environment provides the stage for education, the curriculum provides the script”. Reviews of undergraduate and graduate medical education often emphasize the “formal curriculum” (that is, required courses and explicit educational objectives). Formal curriculum aims to help students develop the core competencies as defined by accreditation agencies, and each educational activity has learning objectives. In addition, the totality of educational sessions must address all the core competencies.

In a study on competency-based medical education in two sub-Saharan African medical schools in Uganda, Elsie Kiguli-Malwadde et al. [4] points to the rising global interest in strengthening human resources for health in the region. More recently, concerns have emerged calling for the transformation of health professionals’ education to better meet the continuing and evolving health needs of communities. In an effort to produce graduates who would better meet the health needs of the Ugandan society, Makerere University in collaboration with John Hopkins University convened stakeholder meetings in the period of 2008 and 2010. Stakeholders included medical school leaders and faculty, students, alumni, healthcare providers, government representatives from the Ministry of Health and Ministry of Education, District Directors of Health, employers of graduates, community leaders and international development partners. From these engagements, the number one recommendation of the Commission on the Education of Health Professionals was the “adoption of competency-based curricula that are responsive to the rapidly changing needs”. Increasing adoption of competency-based medical education (CBME) by health systems and accrediting bodies and medical schools represent an important response to the calls for educational reforms. Governments in sub-Saharan Africa must strengthen their health systems to address emerging disease burdens such as the invasive *Ebola virus* in the region, using strategies to, among others, improve curricula and skills of their workers.

However, contributions to the education literature from the subcontinent are relatively scant. Little is documented on CBME initiatives and the unique circumstances as well as the requirements of implementing CBME in a low-resource environment like sub-Saharan Africa [4]. Western-style university education began in Africa during the colonial occupation [5]. The concept of medical education was established in most African nations in the later part of the colonial period in the 1950s and the 1960s. During the post-colonial period, rapid expansion in medical training was driven by nationalistic sentiments and political pressure [5,6]. The emphasis was to produce a sufficient number of doctors for the needs of the newly independent nations who would be comparable and not “inferior” to the Western-trained doctors. As such, pioneering doctors (future medical educators) were trained at both undergraduate and postgraduate levels in Western universities, and Western curricula were imported for use in African medical schools. At the time when traditional medical training curricula were being imported to Africa, many Western countries were witnessing an increasing demand for a change in line with new pedagogical methodologies [7], replacing learning by rote and accumulation of non-integrated, encyclopedic volumes of knowledge by the promotion of self-directed and lifelong learning skills and personal development [7]. A remarkable global pedagogical shift in medical education has occurred over the last half a century. For many reasons, only a few countries in Africa transformed along with these changes, leading to a future potential for creating quality assurance problems with medical graduates [8].

From literature review, there is almost no evidence that most schools in sub-Saharan Africa have reviewed their curricula since independence. The lack of review is stated as among the factors affecting the performance of professional nurses in Namibia [9]. Institutions that have attempted review have failed to have their curricula address the emerging national needs in the health sector, much less promote effective service delivery among their constituents. The failure of curricula to keep pace with the evolving national health needs, including the chronic disease burden in Africa, has greatly contributed to the deterioration of quality of graduates from these institutions over the years [9]. Kenya is no exception, and a need to investigate the situation in the training institutions to establish the best way forward was necessary in an effort to turn around the health indicators towards achieving Millennium Development Goals as well as universal health coverage by 2030 as one of the United Nation goals for health.

The World Bank Working Paper No. 414 [10] shows that the inadequate capacity of health training institutions in Zambia is a major impediment to ensuring a sufficient flow of health workers into the health labour market as well as ensuring that competency levels are adequate. The paper recommended that a more comprehensive assessment was needed to analyse the bottlenecks in health worker production and performance. In their findings, training capacity has been expanding for nurses more than for medical doctors, and physical capacity of training institutions, while improving, remains inadequate to cater for the demand in the country’s health sector. In the wake of so much need, the review of curricula has never been a priority against other competing needs. As a result, the curricula have failed to address the emerging disease burden in the country.

In the 15 years since independence, Namibia patiently built up a relatively good strategic framework for health policy in the context of government policy as a whole, including strong training arrangements at every level of health staffing and the bringing of human immune deficiency virus/acquired immunodeficiency syndrome (HIV/AIDS) under the strategic umbrella through its National Strategic Plan for HIV/AIDS [10]. Namibia’s major weakness according to the plan is that its curricula do not keep pace with the rise in HIV/AIDS and TB infection; the community counselling service was the only specific response, also under pilot at the time of the study.

The United States Agency for International Development (USAID)-funded CapacityPlus project undertook an assessment of Nigerian midwifery, health technology and nursing schools to better understand the progress and challenges in producing greater numbers of competent and qualified health providers [11]. The assessment revealed that despite steady effort, training institutions faced a number of challenges in their attempts to increase the number and quality of graduates. Quality of graduates is determined by responsive curricula that addresses emerging issues in the health sector [11]. The assessment brought to fore evidence of non-reviewed curricula to meet these requirements. Other inadequacies identified in various areas included the following: admission of unqualified or underqualified students, high student–teacher ratios, limited training and career development opportunities for faculty, infrastructure deficiencies and insufficient learning equipment and materials, including textbooks [11].

The Ministry of Health in Kenya with the support of Capacity Kenya Project, IntraHealth International Inc., in 2009 undertook a performance needs assessment (PNA) of the health training system [12]. The assessment revealed critical gaps and the linkage of the underperformance of Kenya’s health training systems and healthcare service delivery. It was evident that the quality of curricula plays a significant role in determining the quality of graduates churned out of health professional schools [12]. Health workers’ competencies and performance did not conform to requisite standards stipulated by service and other regulatory guidelines, alluding to challenges in quality of training and curricula. Curriculum was not aligned to national priorities with an average of 26% of respondents interviewed at training institutions “not sure” whether the curriculum prepared students adequately to deliver the Kenya Essential Package for Health (KEPH). This is perhaps also an indication that there is a lack of understanding on what the essential package constitutes and how health training institutions should prepare students to deliver these services at health facilities. Respondents were also less confident that the curriculum reflects the current standards developed by appropriate regulatory bodies and the Ministries of Health. Most training institutions did not have a clear policy on curriculum development and review, and lastly, many of the institutions in the healthcare training system lacked sufficient resources, especially in the areas of infrastructure and information and communications technologies (ICTs). From the PNA report, there is need to enquire what the national needs in Kenya are and if the training institutions have a similar understanding with that of the Ministry of Health and stakeholders. It is important to note that emerging diseases such as Ebola and the high prevalence of cancer, diabetes and cross-border diseases, which require surveillance, are not in the training institutions’ pre-service curricula despite the claim that they meet the national needs.

### Case description

IntraHealth’s USAID-funded FUNZOKenya Project is supporting the Government of Kenya (GOK) through the Ministry of Health (MOH) and other stakeholders to address gaps related to training of the health workforce. The goal of the project is to improve access to and quality of health workforce training by supporting an increased number of new health workers, supporting current health worker training needs, strengthening the capacity of training institutions and strengthening regulatory bodies to enhance training demand and quality standardization.

In keeping with the principle of evidence-based practice, the project planned and implemented a baseline assessment of training institutions in nine thematic areas which included the following:

1. Management (financial management, oversight/governance, including government relations with a school);
2. Curricula (theoretical and practical, responsive to needs, regularly updated);
3. Educators or faculty (full/part-time, classroom/clinical recruitment, selection, retention, development);
4. Quality assurance (accreditation of institutions, certification/licensing of graduates);
5. Partnerships and exchange (exchange of faculty/students, partnerships between other schools and with service delivery facilities both public and private);
6. Infrastructure (classrooms, demonstration rooms, laboratories, libraries, computer labs, dormitories, cafeterias, electricity, water, Internet);
7. Materials and equipment (textbooks, teaching/learning materials, computers, anatomical models, simulators, diagnostic equipment, clinical supplies);
8. Clinical practice (variety and appropriateness of sites, mentoring and supervision, infrastructure and equipment); and
9. Students (graduate/postgraduate recruitment, selection, retention).

The project then supported the management of training institutions to prioritize the identified bottlenecks and use them as the entry points for initiating capacity-strengthening efforts. This paper documents the findings of thematic area number 2 above – an assessment of curricula implementation in the institutions in Kenya. The assessment included training institutions from public, private and faith-based organizations (FBOs). The three categories of medical training institutions all use a common curriculum designed by the professional boards depending on the training.

## Methodology

The assessment was a cross-sectional descriptive survey carried out between August 2012 and March 2013 in 14 institutions (3 institutions participated during a pilot stage) purposively selected from among 18 institutions identified for initial collaboration within the project (Additional files 1 and 2). The criteria for selection of these institutions were developed by the project team charged with facilitation of training at the regional hubs. Samples were selected based on regional spread, ownership, size and courses offered, as well as proactive responsiveness and willingness of institutional leaders to collaborate. The assessment collected data from 533 respondents. The assessment questions relating to quality of curriculum sought responses on the following: availability of curriculum guidelines, curriculum responsiveness to institutional mission and regularity of curriculum review as well as involvement of stakeholders in the review process. In addition, the assessment evaluated if the curricula are adequately balanced with respect to theory, demonstration and clinical teaching; had a structure indicating composition, duration and sequence of courses; and prepared students adequately for clinical training. Data was collected using structured questionnaires that also contained open-ended questions. The study was also informed by inputs from a literature review. Among the respondents, 3% were heads of institutions, 15% faculty members, 75% students, 3% clinical site managers and 4% stakeholders – local leaders and government and religious leaders.

The SPSS application was used for quantitative data analysis. Data computation and analysis involved assessing the quality of data, to assist with recoding of categorical variables or grouping variables. Qualitative responses were recorded in Excel sheets then organized by themes and further coded for analysis. Frequencies were used to identify missing cases or few cases. After running frequencies and recoding, cross tabulation was carried out. From the responses, 143 variables were chosen for detailed analysis as these represented the most important variables under each of the thematic areas for diagnosis of bottlenecks.

After analysis, a feedback session was organized with all 14 training institutions. All stakeholders who represented various functions of the institutions were invited to a meeting where findings relating to the nine thematic areas were discussed. The stakeholders were allowed to ask questions, dispute or explain some of the results. Upon reaching a consensus, a list of priorities was drawn and a work plan with the institutions developed to address the identified gaps.

The purpose of the assessment was exploratory in nature, and therefore, the sample size did not represent a significant limitation. The tool was also applied to those whom the institutions could mobilize at the time of the assessment, and therefore, it was possible to reach an adequate number of respondents**.**

The Ministry of Health and the Ministry of Education approved the assessment. The respondents answered the questionnaire voluntarily and signed the consent form in agreement with the provided explanations on the use of information generated from the assessment. No money was given as a motivation. The respondents were also free to discontinue the interview, and respondents’ names would not appear in the report.

## Findings

**Figure 1:**
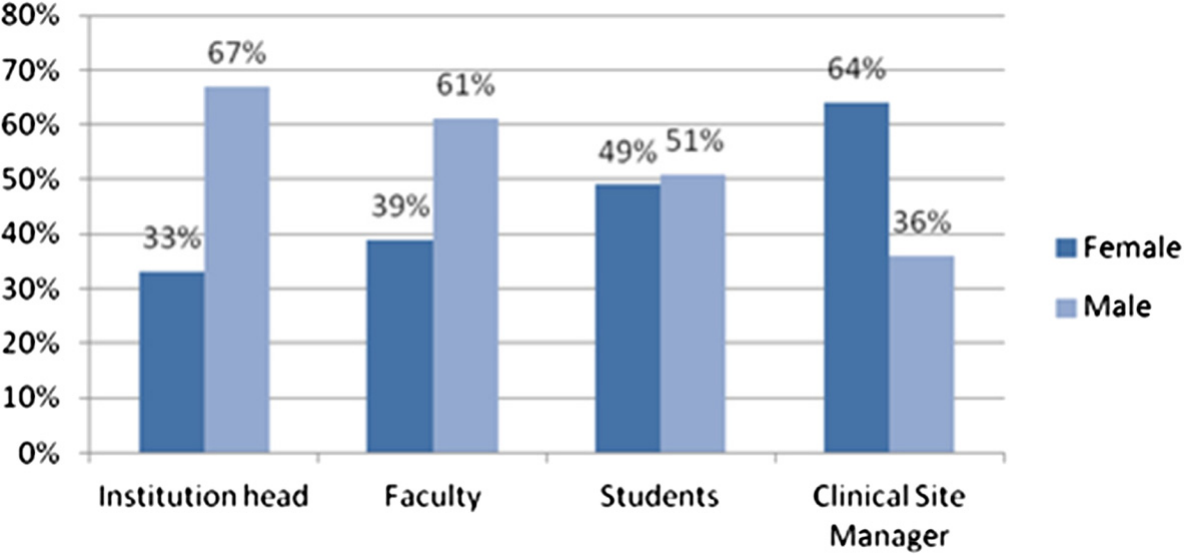
**Characteristics of the respondents.**

This assessment captured the general demographic characteristics of the respondents by gender with the finding showing that more of the male gender have leadership roles in the health training institutions compared to the female gender as indicated in Figure 1 (App). Of the heads of institutions interviewed, 67% were male while only 31% were female. In relation to the faculty, 61% were male while 39% were female. On the other hand, at the clinical sites, there were more female than male managers at 64% and 34%, respectively. The student enrollment showed a balance between the female and male gender with 49% female and 51% male.

Findings from this assessment revealed significant qualitative and quantitative gaps in Kenya’s training institutions including gaps in the curricula despite perception that it responds to national needs. The curricula implementers and student respondents indicated that the current curricula responded to national needs (ranging from a high of about one third private institutions and students to less than one tenth institutional heads) even though stakeholders were not involved in their development. There is need to understand the national needs and if the training institutions’ understanding is similar to that of stakeholders. It is important to note that there are emerging diseases such as Ebola and the high prevalence of cancer and diabetes among others, considered as national needs by Ministry of Health, but not considered by the training institutions in their curricula. In their response, the stakeholders felt that the quality of curricula was inadequate to prepare students for different settings. On average, only one third of respondents felt the quality of curricula prepared students to serve patients in emergency wards. The findings also revealed that curricula do not adequately prepare students for clinical placement in a hospital setting. Similarly, students felt that the quality of classroom delivery of the curricula was inadequate to prepare them for clinical practice. This probably means that students are not quite satisfied with the methodology of curricula delivery. There is a definite disconnect between implementers and stakeholders on the quality of the curricula and its implementation.

## Responsiveness to national health priorities

In terms of ownership of institutions, 81% and 76% of faith-based organizations and private institutions, respectively, perceived their curricula as responsive to the national health needs. Respondents from public training institutions had the lowest perception in this regard with about one third perceiving the curricula as not fully responsive to national health needs. Below are some statements from in-depth interviews that illustrate perceptions on adequacy of the curriculum to the national health priorities:

“The review of our curriculum is overdue. Many diseases have emerged that are challenging to university graduates because they lack teachings to handle them. It’s not in their curricula.” – a faculty from medical training institution.

“Short courses seem to respond to emerging issues than the preservice training because these courses are not part of the curricula. There is need to harmonize the two so that we have a well trained professional to attend to emerging diseases.” – a faculty from medical training institution.

“The curricula addresses national needs but does not adequately prepare us for the practical required in service delivery.” – a student respondent.

Asked whether curriculum adequately prepared students for practical work, a clinical placement manager at a faith-based hospital indicated that, “New methodology of teaching should change to embrace technology. We have a lot of work and we have no time to give proper instructions”.

## Curricula governance structures

**Figure 2:**
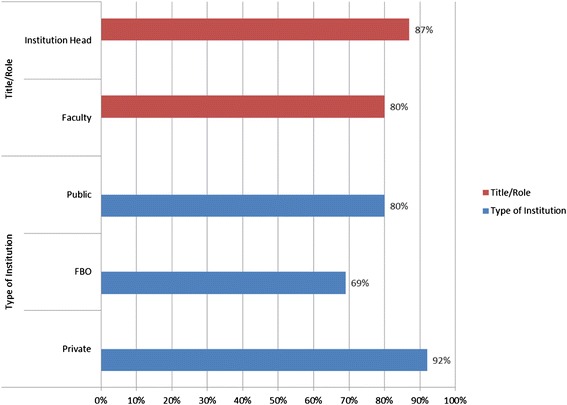
**Perception of written curricula revision guidelines in place.**

The survey questioned the respondents on the existence of written guidelines for curricula review. Respondents from private institutions reported the highest level (92%) of having written guidelines or policy for curricula review. Above 87% of institutional heads and 80% of faculty respondents stated that there is written guidelines for curricula review. Figure 2 (App) provides a graphic view of the data.

**Figure 3:**
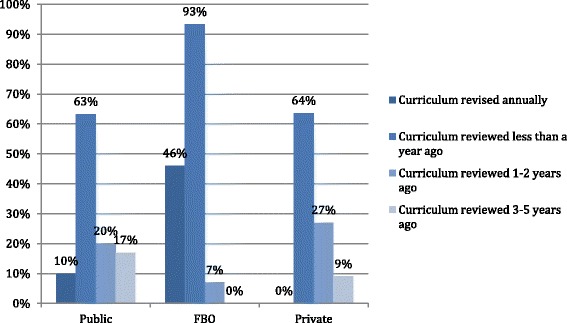
**When curricula was last reviewed.**

In terms of ownership of institutions, none of the private institutions reported to undertake annual curricula reviews. FBOs seem to have a better institutional structure for annual curricula revision with 46% of them reporting annual revision as depicted in Figure 3 (App). The quotes from the in-depth interviews below illustrate perceptions of stakeholders on the standards of curricula framework:

“There is need for a standard curricula framework to guide the curricula reviews for health training courses, because each training institution is coming up with their own things. Some say they have a community-based approach to training of Medicine; others have integrated approach, while others have evidence-based. At the end of the day, they should all be released to the market in Kenya and should offer service delivery. How does this work?” – a stakeholder.

“The institutions do not include us in the curricula development, yet we are consumers and stakeholders in health. How can they claim their curricula meet national needs? There has to be a consensus about this.” – a stakeholder.

## Stakeholder involvement in curricula development and review

The assessment findings show that only 29% of institution heads and 35% of faculty members perceived high stakeholder involvement in their curricula review process. Data seems to suggest that medical training institutions in Kenya need to work towards having a higher involvement of stakeholders in their curricula design and development process to achieve educational goals defined for medical students.

**Figure 4:**
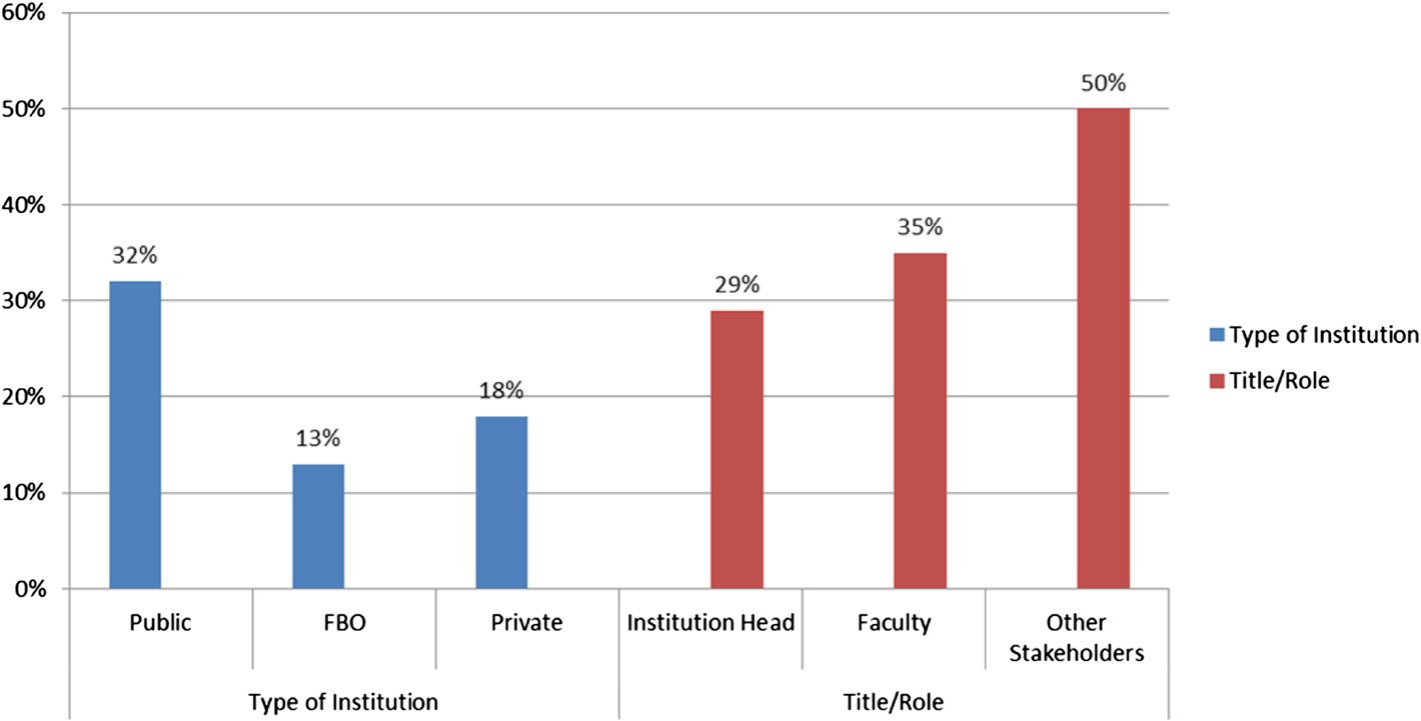
**Perception of high stakeholder involvement in curricula development.**

A third of respondents from public institutions stated that there was “high” stakeholder involvement for curricula development. Only 13% of FBO respondents and 18% of private institution respondents perceived high stakeholder involvement in their curricula development (see Figure 4). In relation to involvement in curricula review, 50% of the other stakeholders (clinical preceptors) reported a high level of involvement in curricula review.

The respondents were also asked to rate their satisfaction on the quality of training curricula that prepares students for different settings. Data from the assessment in Table 1 (App) suggests that most of the stakeholders felt that the quality of curricula was inadequate in preparing students for different settings. On average, only one third of respondents felt the quality of curricula prepared students to serve patients in emergency wards. The findings show that curricula are not adequate to prepare students for clinical practice in a hospital setting. In respect to classroom teaching, only 41% of students felt that the quality of classroom delivery of the curricula was adequate to prepare students for their clinical practice. It probably means that students are not quite satisfied with the methodology. The quality of training was measured against the threshold of greater than 75% as being “adequate”. Data also suggests a deep dissatisfaction with the quality of training currently provided as none of the respondents felt that the training in any of these settings was adequate to prepare students.

**Table 1:** **Perception of adequacy of quality of training in preparing students in the listed settings**

**Learning setting**	**Institutional head (%)**	**Faculty (%)**	**Student (%)**	**Clinical site manager (%)**
Classroom	60	65	41	NA
Skills lab	57	33	27	NA
Clinical practice sites	40	43	45	31
Provincial hospital	25	41	39	30
District hospital	43	37	34	27
Outpatient clinic	29	36	39	46
Health centre	47	36	37	44
Maternity ward	42	34	44	39
Emergency ward	21	27	38	40
Community service	43	42	41	40

Note: all percentages less than 75% have been rated as inadequate.

The survey also sought views on best practices and improvements on curricula in these institutions. Table 2 (App) provides the feedback from different stakeholders. Almost all the stakeholders recognized that timely review and updating of curricula should reflect health needs as a definite area for improvement. Students (32%) felt the need for more learning resources both in terms of teachers as well as infrastructure for effective content delivery.

**Table 2:** **Best practices and suggestions offered by respondents to improve curricula content, revision and delivery**

	**Institutional head (%)**	**Faculty (%)**	**Clinical site manager (%)**	**Student (%)**	**Other stakeholders (%)**
Timely review and update of curricula to include current issues in health is needed	33	33	11	20	27
Aligning theory and practice by increasing more practical learning	22	2	0	22	46
Enhance participation of all stakeholders including students	22	40	78	20	18
Community needs addressed in curricula	22	9	0	3	9
Available learning resources both human and infrastructure	11	9	0	32	27
Level of lecturers training and student entry level	11	7	11	5	
Technology to be integrated	0	9	0	4	18

The above highlights and more reveal a consensus on the need for the government and stakeholders to pay attention to the review of the curriculum offered in Kenya.

## Discussion

As indicated in the standards set by the World Federation for Medical Education (WFME) [13], programmes in education should ensure that the curriculum, its design and proposed instructional methodology lead to achievement of the requisite competencies for clinical practice. The study assessed aspects of curriculum revision, responsiveness of the curriculum to national health priorities, updating of curricula and involvement of stakeholders in curricula revisions.

A curriculum must strike a balance with respect to theory, demonstration and clinical teaching. In addition, it should have a structure indicating composition, duration and sequence of courses and with capacity to adequately prepare students for clinical training.

The findings of this study identify major gaps in the curricula development and review in health professional training institutions in Kenya. Timely review and update of curricula to include current issues in health is needed. Additionally, curricula should align theory and practice by increasing more practical learning, enhancing participation of all stakeholders, including students, and that curricula should address community needs. In the study, there is an indication of no developed plans to audit not only clinical placement but also competencies of clinical instructors. It also reveals a need to form committees to review curricula and to integrate a dissemination mechanism for the policies and respective guidelines in addition to publishing the official curricula review standards. This is in line with a study on competency-based medical education in two sub-Saharan African medical schools in Uganda, where Makerere University and John Hopkins University collaborated and convened stakeholder meetings from 2008 to 2010 [4] in an effort to produce graduates who would better meet the health needs of the Ugandan society. Stakeholders included medical school leaders and faculty, students, alumni, healthcare providers, government representatives from the Ministry of Health and Ministry of Education, District Directors of Health, employers of graduates, community leaders and international development partners. The Commission on the Education of Health Professionals resolved to the “adoption of competency-based curricula that are responsive to rapidly changing needs”. Increasing the adoption of CBME by health systems, accrediting bodies and medical schools represents an important response to the calls for educational reform. It affirmed that governments in sub-Saharan Africa must strengthen their health systems, adopting one such strategy as improving curricula and skills of their workers.

The World Bank Working Paper No. 414 [10], following a comprehensive assessment to analyse the bottlenecks in health worker production and performance in more detail in Zambia, stated that the lack of review led to the failing of curricula to address the emerging disease burden and population of its country.

According to the findings, few or no standing committees exist in the various institutions for review of curricula that integrate membership of clinical instructors/preceptors in order to enrich integration of emerging issues in service delivery, thereby enhancing more responsive curricula. Institutions supported the establishment of joint committees for curriculum reviews incorporating clinical instructors and preceptors at clinical placement facilities and to ensure dissemination of relevant policies and guidelines. They also advised on strengthening of curriculum development and review and governance to ensure quality of content and delivery. The stakeholders involved in the medical training of students and inputs of the students themselves are integral to development and design of curricula. According to the WFME, the school must have a policy on student representation and appropriate participation in the design, management and evaluation of the curricula and in other matters relevant to students [13]. The CBME study has demonstrated that an all-inclusive curricula review and development can effectively respond to emerging national needs.

Findings also show that no standard curricula framework exists to guide curricula reviews for health training courses. This creates *ad hoc* reviews by institutions that are likely to fizzle out with changes of management. Institutions should take advantage of policies and frameworks from regulatory bodies to guide curricula review. The PNA report [12] equally raised concerns of non-adequacy of curricula to prepare students to adequately deliver the KEPH. It is possible that institutions have taken steps to address curricula needs based on the inputs from the PNA report, although there is room for improvement, especially in public health institutions.

The findings are silent as to the level of involvement of the MOH in the curricula review process. Integration of MOH in the curricula review committees would be important in updating faculty on emerging issues in policy as well as emerging disease patterns and new management protocols. Although integrating preceptors in curricula reviews has the potential to strengthen performance and competencies of graduating health workers, involving the MOH would give an overall picture of the holistic prevailing needs in the health sector. The MOH had identified KEPH as the minimal standard for delivery of quality primary health services for all Kenyans [14]. As such, it is imperative that the curricula be revised regularly to be responsive to the needs of national health priorities and that the curricula prepare students to deliver the KEPH. There is need for a common understanding of the national needs collectively defined by the MOH, training institutions and other key stakeholders to have training informed by an agreed framework. Additionally, Kenya, like other countries in the region, faces practical challenges with regard to emerging cross-border diseases such as the *Ebola virus* and a high prevalence of cancer and diabetes, among others, that require surveillance, which is not in the training institutions’ curricula but currently addressed by in-service trainings.

## Conclusion

The findings of this assessment provide evidence that review of curricula to meet the national needs is critical to the quality and relevance of training of the health workforce in Kenya. Although it defines the direction of training institutions and quality of products from these institutions, it lacks in prominence and has minimal attention. Kenya cannot meet the required number and quality of its health workforce without the training institutions. The training institutions, on the other hand, cannot meet demand unless they have the capacity to do so. Standard policy on training around the review of curricula is necessary as harmonization of all curricula of training institutions would ensure the quality of trainings and eventual quality service delivery. Evidence from this assessment shows that if the health sector is to turn around its health indicators to meet the Millennium Development Goals related to health, and achieve universal health coverage by 2030, more attention and support to training institutions to address the bottlenecks highlighted in the assessment is paramount. Review of the training curricula should be the first priority as it defines and determines the requirements for the trainings and should be directed by the Ministry of Health’s Human Resource Development (HRD) unit. The capacity to sustainably address the challenge of health worker shortage demands the inclusiveness of all stakeholders in strategy design and implementation.

## Recommendations

- Government and health training institutions should prioritize curricula development and review in line with health sector needs.
- There is need to establish joint committees for curriculum reviews to incorporate clinical instructors and preceptors at clinical placement facilities, with dissemination plans for relevant policies and guidelines to all.
- Curriculum development and review and governance should be strengthened to ensure quality of content and delivery.

## Additional files

Additional file 1:
**Bottleneck Assessment tools- School/Program Head/Faculty-General Information.**
Additional file 2:
**Bottleneck Professional Assessment tool- Key Informant tool.**

## Abbreviations

CBME: Competency-based medical education
FBO: Faith-based organization
GOK: Government of Kenya
HIV/AIDS: Human immune deficiency virus/acquired immunodeficiency syndrome
HRD: Human Resource Development
KEPH: Kenya Essential Package for Health
MOH: Ministry of Health
PNA: Performance needs assessment
SPSS: Statistical package for social sciences
TB: Tuberculosis
USAID: United States Agency for International Development
WFME: World Federation for Medical Education
WHO: World Health Organization
