# Supplementary material for: Assessment of quality and relevance of curricula development in health training institutions: a case study of Kenya
Source: Hum Resour Health. 2015 Aug 13;13:67. doi: 10.1186/s12960-015-0048-9 (PMC4535832; doi:10.1186/s12960-015-0048-9)
Supplement: Additional file 1: — Bottleneck Assessment tools- School/Program Head/Faculty-General Information. [file 12960_2015_48_MOESM1_ESM.doc]

**
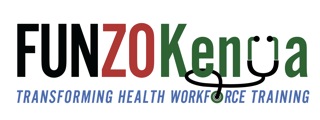

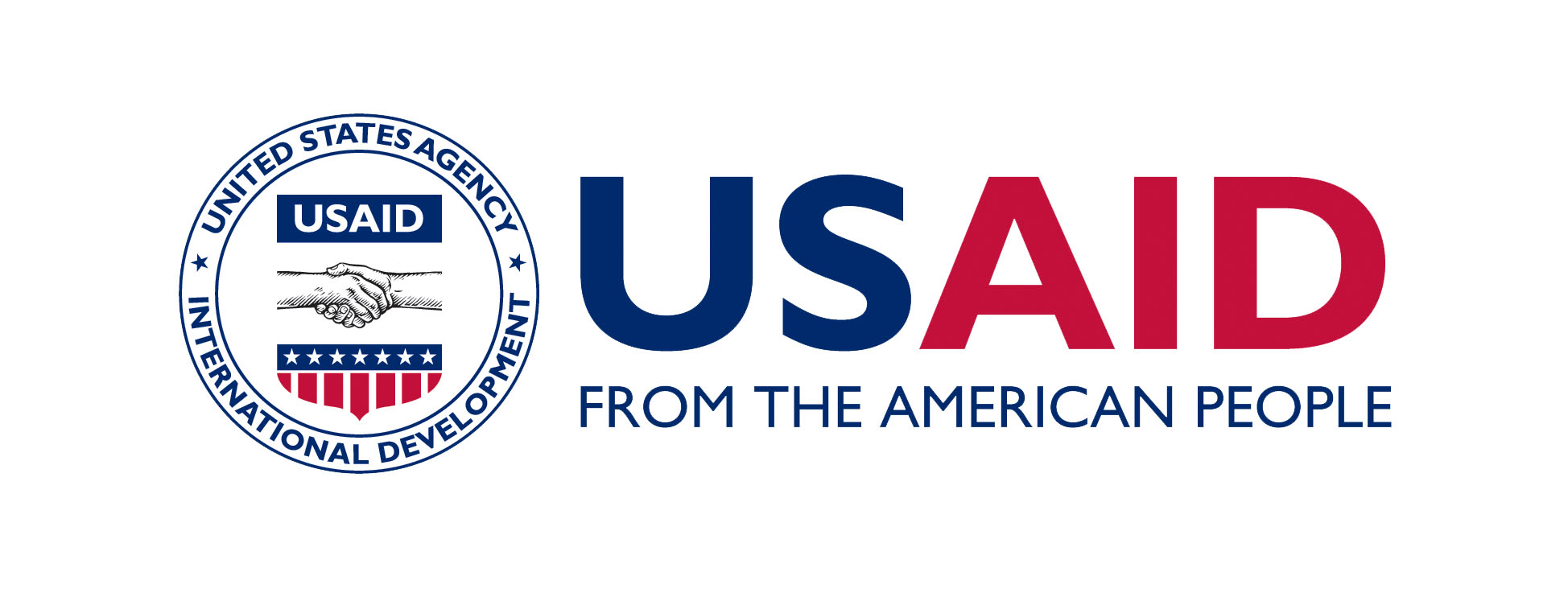

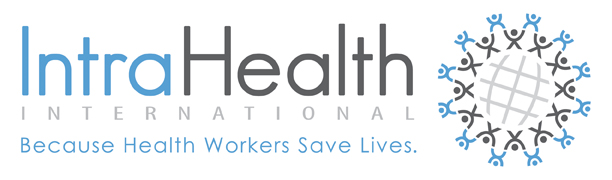
Bottlenecks Assessment Tool:** Tool Code: ___|___|____|____|____

**School/Program Head/Faculty:**

**Ownership:**

**Date:**

# General Information

1. Gender of respondent: 1) Female 2) Male 2. Age …………….Yrs
2. State your academic Role(s)/Responsibility (ies): ………………………………………………………………………………
3. What is the total number of years served in academic post ..........
4. State your highest academic qualification attained……………………………………………………………………….
5. Do you have formal training in pedagogy (medical education)? 1)No 2) Yes
6. If yes, classify the training: (CIRCLE ONE) 1. Short course without certification 2. Short course with certification 3. Formal/recognized course with certification from regulatory body
7. If yes, how many years since attending (last) course? ….............yrs
8. Do you offer E-/Distant learning? (Head) 1. Not sure 2. No 3. Yes
9. Do you have weekend/Evening Classes? (Head) 1. Not sure 2. No 3. Yes
10. What is total Student population ………..
11. Please enter details of academic programs offered in your institution

| Name/type of program | Type of qualification earned (e.g. certificate, diploma, degree, Masters, PhD) | # of students admitted in last academic year:  F: M | % of students registered for final exam who passed last academic year:  F: M | % of students certified by professional body:  F: M | % of students who dropped out  F: M | % of students who repeated:  F: M |
| --- | --- | --- | --- | --- | --- | --- |
|  |  |  |  |  |  |  |
|  |  |  |  |  |  |  |
|  |  |  |  |  |  |  |
|  |  |  |  |  |  |  |
|  |  |  |  |  |  |  |
|  |  |  |  |  |  |  |
|  |  |  |  |  |  |  |
|  |  |  |  |  |  |  |
|  |  |  |  |  |  |  |
|  |  |  |  |  |  |  |

1. Has your school identified any programs it wishes to scale up but experiencing some challenges?

1) No 2) Yes ….Specify up to three: …………………………………………………………………..

# Infrastructure, Equipments, Materials & Supplies

1. When was the last facility audit conducted? (Completed years)………………………

Please provide capacity rating for the following infrastructures at your school?

| **Infrastructure Elements** | **Capacity Rating:**  0=Not available; 1=< 50% of requirement; 2= 50-75% of requirement; 3= >75% requirement; DK=Don’t Know |
| --- | --- |
| Faculty offices |  |
| Administrative offices/staff rooms |  |
| Classrooms/lecture rooms |  |
| Libraries |  |
| students Study/meeting rooms |  |
| Clinical laboratories |  |
| Skills labs/demonstration rooms |  |
| Mutli-purpose hall |  |
| ICT |  |
| Accommodation/Dormitories |  |
| Student Chairs and Tables |  |
| Audiovisual teaching aids |  |
| Anatomical models |  |
| Standard Text books |  |
| Reference books |  |
| Electronic teaching and learning materials |  |
| Consumable materials/Supplies |  |

Please rate the availability and quality of the following elements on a scale of 1-5

| **Element** | **Rating:**  1=Very poor; 2=poor; 3=Average; 4=good; 5=Very good |
| --- | --- |
| 1. Internet Connectivity |  |
| 1. Electricity |  |
| 1. Ventilation |  |
| 1. Safe water |  |
| 1. Work place Safety & Security |  |
| 1. Other teaching aids |  |
| 1. Psychosocial Working environment |  |
| 1. Overall rating of access & Quality of Educational Resources |  |

1. Do you own a personal computer or laptop? 1. No 2. Yes
2. Is there a written policy on maintenance & repair of equipments? 1. Not 2. No sure 3. Yes

# Curriculum

1. Are the competencies to be learnt clearly stated and aligned to current societal needs?
   1. Yes but very limited 2) Partially 3) Yes clearly & Fully
2. Please rate the functional capacity of curriculum development committee in the school:

0) None exists 1) Only partially functional 2) Fully Functional

1. To what extent does the curriculum committee have **authority** of curriculum **design** and **resources** for implementation? 1) Very limited 2)Partial 3)Full authority
2. Please rate stakeholder involvement in the school’s curriculum review process:

0) Nil, 1) Very low, 2) Below average, 3) Average, 4) Above average, 5) Very high/fully involved

1. Does the school have **written** curriculum review Guidelines? 1. Don’t know 2. No 3. Yes
2. Please rate the level of compliance with Curriculum Review Guidelines:

1) Very low compliance, 2) below average compliance, 3) average compliance, 4) above average compliance, 5) Full compliance

1. How would you rate the training curriculum in preparing students in the settings below?

| **Teaching/Learning settings** | **Quality**  0=No opinion, 1=Very Poor, 2=Poor, 3=Average, 4=Good, 5=Very good, |
| --- | --- |
| Classroom/theoretical teaching |  |
| Skills lab/demonstration room |  |
| Clinical practice sites in general |  |
| Hospitals (L 4-6) |  |
| Primary care Settings (L 1-3) |  |
| Maternity ward/service |  |
| Emergency ward/care |  |
| Administration & Management |  |
| Professionals teamwork groups |  |

1. Please rate the practice of inter-professional and teamwork approaches in curriculum delivery

1=Very limited; 2=Limited; 3=Moderate; 4=High/Good; 5=Very High/Good

1. Do you collaborate with other regional schools on training curriculum?

0. No 1. Don’t know 2. Yes

1. List main recommendations for curriculum improvement:…………………………………………………………………. ……………………………………………………………………………………………………………………………………………………………

# Faculty

Please state the following faculty statistics/information (Only Head to complete):

|  | Clinical subjects  F M | Basic sciences  F M | Clinical instructors  F M | Total  F M |
| --- | --- | --- | --- | --- |
| Total No. of staff, incl. part time |  |  |  |  |
| What is the % shortfall in faculty |  |  |  |  |

1. (Head): What percentage of faculty is part time? (Write 0 if none) ……………….
2. (Head): Give estimate of the classroom teacher/faculty: student ratio? ………………
3. (Head): Give estimate of the clinical instructor: student ratio? ……………………..
4. Please rate the use of ICT in teaching/training:

0) Never, 1) Rarely, 2) Occasionally, 3) half the time, 4) Most of the time, 5) All the time

1. Does the institution have a policy for balanced recruitment of both male and female educators on a 50% by 50% basis? 0. Don’t know 1.No 2. Yes
2. Does your school have a policy or procedure that supports pregnant educators, or educators with young children, and generally encourage female retention? 0. Don’t know 1.No 2. Yes
3. How many cases of gender violence/harassment were reported in the school in last 1 yr?

0. None 1. 1or2 2. 3-5 3. 5-10 4. >10

1. Do you have written policy on sexual harassment of teachers? 0. Don’t know 1.No 2. Yes
2. Is it **mandatory** for faculty to participate in regular CPD? 0. Don’t Know 1. No 2. Yes.
3. How would you rate the **regular participation** of faculty in CPD?

1. Very low/poor 2. Low/poor 3. Average 4. Good 5. Very regular/high

1. Is there a regularized CPD program for teachers/instructors? 0. Don’t Know 1. No 2. Yes
2. Please rate the availability of Research Opportunities for faculty?

1) Very few/limited, 2) Limited, 3) average, 4) Above average, 5) Very high/plenty

1. Do you have dedicated research budget vote? 1. Don’t Know 2. No 3. Yes
2. If yes please give an estimate of the value annually in Ksh. ………………………….
3. How would you rate/describe staff turnover in the school?

1. Very low turnover 2. Low turnover 3. Average 4. High turnover 5. Very high turnover

1. Please list maximum of three most common reasons for faculty high turnover: …………………….
2. Rate the experiences sharing between classroom teachers and clinical instructors at clinical facilities
3. Nil, 1) Very limited, 2) below average, 3) Average, 4) Above average, 5) very frequent/high
4. Which performance evaluation tools are used in the school? ............................................

……………………………………………………………………………………………………………………………………………………

1. Please list **up to five** specific knowledge and skills gap areas/topics for which the faculty could benefit from additional training: ………………………………………………………………………………………………………

…………………………………………………………………………………………………………………………………………………………………

1. Please List key recommendations for faculty improvement: ………………………………………………………… ……………………………………………………………………………………………………………………………………………………….

# Management

1. To what extent has the educational Program Mission & Objectives been disseminated & displayed? 1)Limited 2)Partially 3)Fully/extensively
2. What is the proportion of female staff in the school’s management system?

1. Upto one third(1/3) 2. >1/3^rd^ to Half 3. >Half to 2/3^rd^ 4. > 2/3^rd^

1. What is the % shortfall in administrative staff? ………
2. Do you have a Faculty Development Policy? 0. Don’t Know 1. No 2. Yes
3. If yes give a summary description: ……………………………………………………………………………………………

…………………………………………………………………………………………………………………………………………………..

1. Please rate the following elements of management on a scale of 1-5:

| **Element** | **Rating:**  1=Strongly disagree; 2=Disagree; 3=Neutral; 4=Agree; 5=Strongly agree |
| --- | --- |
| 1. Staff have Realistic Workload |  |
| 1. Staff get Supportive supervision |  |
| 1. Staffs have Clear Roles, Responsibilities and Authority/Autonomy |  |
| 1. There is a mechanism for Recognition and Reward of highly performing staff |  |
| 1. There is Adequacy of Financial Resources for the academic programs |  |
| 1. There is Effective Management & Leadership of the academic programs |  |
| 1. Staffs have adequate opportunities for Career Progression |  |
| 1. There is Clear Communication & Information Channels and Access |  |
| 1. The Hiring, management and performance appraisal System is fair & Transparent |  |
| 1. The institution has a well functioning Governance Board/Council |  |

1. What kind of management evaluation systems/tools do you use? …………………………………………

………………………………………………………………………………………………………………………………………………..

1. Do you have a formal system of engaging alumni? 0. Don’t Know 1. No 2. Yes
2. Do you have automated database for tracking student grades? 0. Don’t Know 1. No 2. Yes
3. What is the level of compliance in student enrollment to regulatory standards?
4. Under-enrollment, 2) appropriate enrollment, 3) Over-enrollment
5. Are you aware of standards for administrative staff to student ratio? 0. Not Sure 1. No 2. Yes
6. If yes, what is the level of conformity to those standards? 1) Very low compliance, 2) below average compliance, 3) average compliance, 4) above average compliance, 5) Full compliance
7. Does the school have a funding system for needy students? 0. Don’t Know 1. No 2. Yes
8. If yes, how many were supported in last one year............................................
9. What is the total annual budget for the health sciences programs last year? ……………..
10. What is the average annual student fee? …………………
11. What are the main sources of funds for running the programs and proportion of each?

1) Government …2) Out of Pocket Fees 3) Funded Research/Consultancy …. 4)Donations …5)Others..

1. Please rate the quality of maintenances of school infrastructures & Equipments:

1) Very poor, 2) below average, 3) Average, 4) Good/Above average, 5) Very good

1. Do have regular inclusive engagement of all stakeholders in decision making? 1) No 2)Yes
2. Does the school have all the **written** Policies and Guidelines relevant to educational programs as prescribed by the relevant regulatory bodies?

1) Yes but only a few 2)Yes, Half of them 3)Yes most of them 4)Yes all of them

1. For those missing, please indicate which key ones: ………………………………………………………………….
2. Please list main recommendations for improving the school’s management: ………………………………………………………..…………………………………………………………………………

# Student Enrollment & Retention

1. What proportion of students who apply and qualify actually get admitted?

1. <10% 2. 10-25% 3. 26-35% 4. 36-50% 5. 51-75% 6. >76%

1. What proportion of student population is from rural settings? ..........%
2. Is there regional balance policy in student enrollment? 0. Don’t Know 1. No 2. Yes
3. Is there a gender balance policy in student recruitment? 0. Don’t Know 1. No 2. Yes
4. Please rate fairness and transparency of student enrollment and admission process:

1) Very unfair; 2) Unfair; 3) Average; 4) Fair/open; 5) Very Fair/open

1. Please rate counseling and support for students for the elements below

| **Student support area/element** | **1) Very poor; 2) poor; 3) Average; 4) Good; 5) Very Good** |
| --- | --- |
| Academic Counseling |  |
| Personal needs |  |
| Social needs |  |
| Financial needs |  |
| Confidentiality of student information |  |
| Resources to facilitate student support |  |
| Aggregate/General (Average of all above) |  |

1. Do you know of any student who was a victim of gender-based violence in the school?

0. Don’t Know 1. No 2. Yes

1. Is there support available for victims of gender-based violence? 0. Don’t Know 1. No 2. Yes
2. Is there a written student sexual harassment school policy? 0. Don’t Know 1. No 2. Yes
3. Is students dropping out before completion ‘big’ problem?  0. Don’t Know 1. No 2. Yes
4. If yes, what do you think is/are the reason(s)? (CIRCLE ALL THAT APPLY)

1)Low interested, 2)School fees, 3)Get married, 4)Get pregnant, 5)Poor/bad course, 6)Too much information, 7)Too difficult course, 8)School too far, 9)Strict rules, 10)Other (Specify)…………………

1. Do you have a policy addressing students who have physical disability? 1)Yes 2)No
2. Please rate the Representation/Participation of students in the schools decision making:

1) Very limited; 2) Limited; 3) Average; 4) Good/High; 5) Very Good/High

1. What are the main recommendations regarding students in health training programs? ………………………………………………………………………………………………………………………………………

# Clinical Placement & Practice

1. Are you aware of clinical placement guidelines? 0. Don’t Know 1. No 2. Yes
2. If yes, what is the level of conformity to clinical placement guidelines?

1) Very low compliance, 2) below average compliance, 3) average compliance, 4) above average compliance, 5) Full compliance

1. How would you rate the availability of clinical instructors/preceptors?

1=Very Inadequate; 2=Inadequate; 3=Average; 4=Adequate; 5=More than adequate

1. Do clinical instructors have a **regularized** CPD program? 0. Don’t Know 1. No 2. Yes
2. How would you rate the availability of infrastructure at clinical placement sites?

1=Very Inadequate; 2=Inadequate; 3=Average; 4=Adequate; 5=More than adequate

1. How would you rate the availability of Equipments & Materials at clinical placement sites?

1=Very Inadequate; 2=Inadequate; 3=Average; 4=Adequate; 5=More than adequate

1. What mechanisms does the school have for securing quality of clinical placement sites? ………………………………………………………………………………………………………………………………………………………….

# Quality Assurance & Standards

1. When was the last accreditation visit made by the accrediting body? ………………….
2. Are you aware of facility regulatory standards for medical training? 0. Don’t Know 1. No 2. Yes
3. If yes please what is the level of compliance in general, and in following aspects?

| **Aspect of standard** | **Compliance level**  1=Very poor; 2=Poor; 3=Average; 4=Good/Above average; 5=Very good/Full |
| --- | --- |
| QA in General |  |
| Physical infrastructure |  |
| Equipments and supplies |  |
| Staffing |  |

1. Does the school have written policies on quality assurance? 0. Don’t Know 1. No 2. Yes
2. If yes, please describe ………………………………………………………………………………………………………………..

# Partnership, Exchange and Networking (PEN)

1. Does your school have any partnerships or exchange programs with other schools?

0. Don’t Know 1. No 2. Yes

1. What types of PEN activities are common in your school/program? (Write 0 if none)

…………………………………………………………………………………………………………………………………………

1. Number of exchange in last one year: Students = …………Faculty = …………
2. How would you rate the engagement of the school in academic networks?
   1. Nil, 1) One or two networks, 2) Several networks, 3) Many networks
3. List the main recommendations on PEN for the school: Include ………………………………………………………………………………………………………………………………………………..

……………………………………………………………………………………………………………………………………………………….
