# Supplementary material for: Assessment of quality and relevance of curricula development in health training institutions: a case study of Kenya
Source: Hum Resour Health. 2015 Aug 13;13:67. doi: 10.1186/s12960-015-0048-9 (PMC4535832; doi:10.1186/s12960-015-0048-9)
Supplement: Additional file 2: — Bottleneck Professional Assessment tool- Key Informant tool. [file 12960_2015_48_MOESM2_ESM.doc]

**
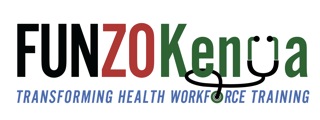

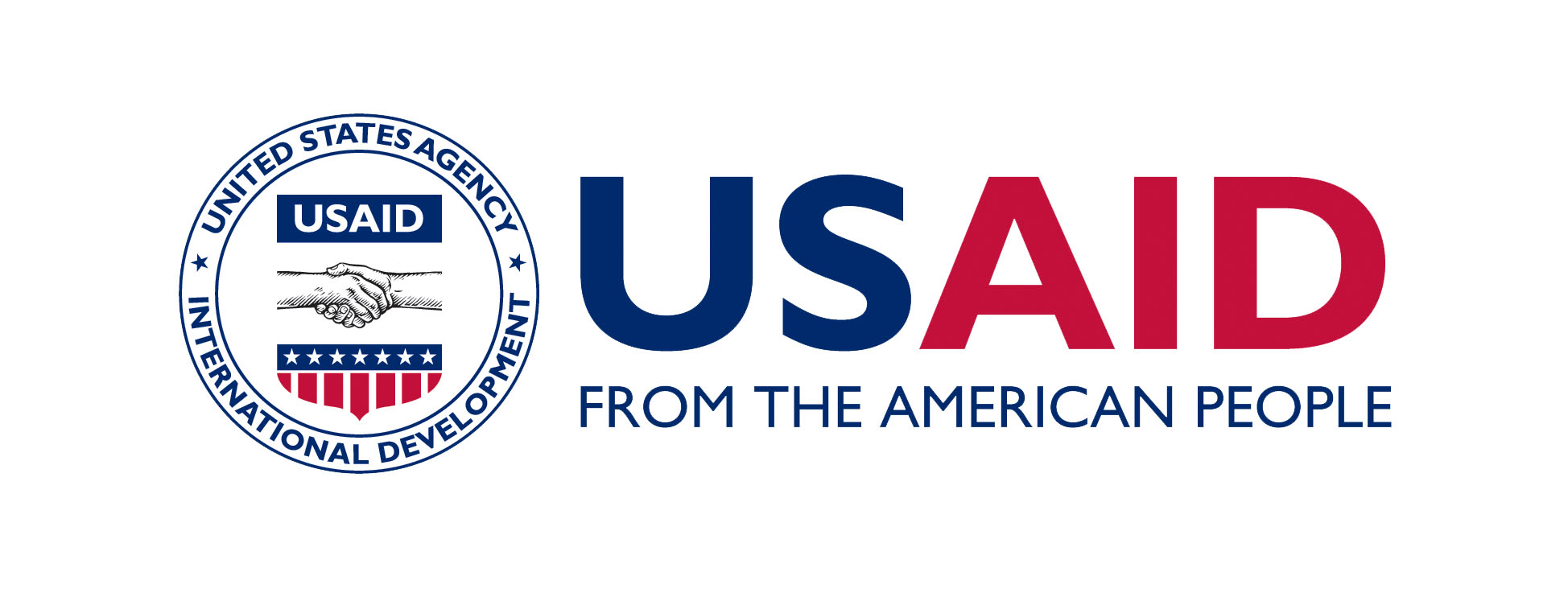

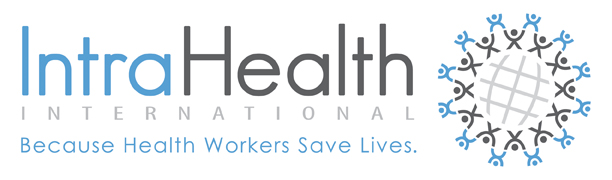
Bottlenecks Assessment Tool:** Tool Code: ___|___|____|____|____

**Key Informant Tool**

# General Information

1. Name of institution ……………………………. Ownership 1. Public 2. FBO 3. Private
2. Gender of respondent: 1) Female 2) Male 5. Age …………….Yrs
3. Which of the following Roles/Responsibilities are relevant to you? (Tick all that apply):

1) School Governance/Leadership 2) Government Official 3) Private sector 4) Community stakeholder 5) Others..(Specify) ………………………………………………………………..

# Infrastructure, Materials, Equipments & Supplies

Please provide capacity rating for the following infrastructures at your school?

| **Infrastructure Elements** | **Capacity Rating:**  0=Not available; 1=< 50% of requirement  2= 50-75% of requirement; 3= >75% requirement; DK=Don’t Know |
| --- | --- |
| Faculty offices |  |
| Administrative offices/staff rooms |  |
| Classrooms/lecture rooms |  |
| Libraries |  |
| students Study/meeting rooms |  |
| Clinical laboratories |  |
| Skills labs/demonstration rooms |  |
| Mutli-purpose hall |  |
| ICT |  |
| Accommodation/Dormitories |  |
| Student Chairs and Tables |  |
| Audiovisual teaching aids |  |
| Anatomical models |  |
| Standard Text books |  |
| Reference books |  |
| Electronic Teaching/Learning materials |  |

# Curriculum

1. Do you believe that the medical courses offered by the school are relevant to the current community health needs?
   1. Yes but to very limited extent 2) Yes, Partially 3) Yes, Fully
2. Please rate stakeholder involvement in the school’s curriculum review process:

0) Nil, 1) Very low, 2) Below average, 3) Average, 4) Above average, 5) Very high/fully involved

How would you rate the training curriculum in preparing students in the settings below?

| **Teaching/Learning settings** | **Quality**  0=No opinion, 1=Very Poor, 2=Poor, 3=Average, 4=Good, 5=Very good, |
| --- | --- |
| Classroom/theoretical teaching |  |
| Skills lab/demonstration room |  |
| Clinical practice sites in general |  |
| Hospitals (L 4-6) |  |
| Primary care Settings (L 1-3) |  |
| Maternity ward/service |  |
| Emergency ward/care |  |
| In Health service administration & Management |  |
| Working within team of health professionals |  |

1. List main recommendations for curriculum improvement:…………………………………………………………………. ……………………………………………………………………………………………………………………………………………………………

# Faculty

1. What is your view on adequacy of teachers/instructors?

1) Very Inadequate 2) Inadequate 3) Fairly adequate 4) Above average 5) Very adequate

1. How would you rate/describe staff turnover in the school?

1. Very low turnover 2. Low turnover 3. Average 4. High turnover 5. Very high turnover

1. Please List key recommendations for faculty improvement: ………………………………………………………… ………………………………………………………………………………………………………………………………………………………..

# Management

1. To what extent has the educational Program Mission & Objectives been disseminated & displayed? 1)Limited 2)Partially 3)Fully/extensively
2. Please rate the quality of maintenances of school infrastructures & Equipments:

1)Very poor, 2) below average, 3) Average, 4) Good/Above average, 5) Very good

1. What is the level of security and safety?

1)Very poor, 2) below average, 3) Average, 4) Good/Above average, 5) Very good

1. Rate the level of engagement of all stakeholders in decision making?

1) Very Low 2) Low 3) Average 4) High 5) Very High

1. How would you rate compliance of the school with standard educational policies?

1) Very Low 2) Low 3) Average 4) Good 5) Very Good

1. Please list main recommendations for improving the school’s management: ………………………………………………………..……………………………………………………………………..

# Student Enrollment & Retention

1. Is there regional balance in student enrollment? 0. Don’t Know 1. No 2. Yes
2. Is there a gender balance in student recruitment? 0. Don’t Know 1. No 2. Yes
3. Do you believe there is a big problem of students dropping out of school before completion?

0. Don’t Know 1. No 2. Yes

1. If yes, what do you think is/are the reason(s)? (Circle all that apply)

1)Low interested, 2)School fees, 3)Get married, 4)Get pregnant, 5)Poor/bad course, 6)Too much information, 7)Too difficult course, 8)School too far, 9)Strict rules, 10)Other (Specify)…………………

Does the school policy address students with physical disability? 0. Don’t Know 1. No 2. Yes

1. What are the main recommendations regarding students in health training programs? ………………………………………………………………………………………………………………………………………

# Clinical Placement & Practice

1. Do you believe the clinical placement practices adequately prepare students for real life practice?

0. Don’t Know 1. No 2. Yes

# Quality Assurance & Standards

Please rate the level of compliance in general in following aspects of QA?

| **Aspect of standard** | **Compliance level**  1=Very poor; 2=Poor; 3=Average; 4=Good/Above average; 5=Very good/Full |
| --- | --- |
| QA in General |  |
| Physical infrastructure |  |
| Equipments and supplies |  |
| Staffing |  |

# Partnership, Exchange and Networking (PEN)

1. Does your school have any partnerships or exchange programs with other schools?
   1. Yes 2. No 3. I don’t know
2. What types of PEN activities are common in the school/program? (Write 0 if none)

…………………………………………………………………………………………………………………………………………

1. How would you rate the engagement of the school in community work?
   1. Nil, 1) One or two networks, 2) Several networks, 3) Many networks
2. List the main recommendations on PEN for the school: Include ………………………………………………………………………………………………………………………………………………..

……………………………………………………………………………………………………………………………………………………….

***Thank you very much!***
